# Recognition of Bathroom Activities in Older Adults Using Wearable Sensors: A Systematic Review and Recommendations

**DOI:** 10.3390/s21062176

**Published:** 2021-03-20

**Authors:** Yiyuan Zhang, Ine D’Haeseleer, José Coelho, Vero Vanden Abeele, Bart Vanrumste

**Affiliations:** 1 KU Leuven, e-Media Research Lab, 3000 Leuven, Belgium; ine.dhaeseleer@kuleuven.be (I.D.); vero.vandenabeele@kuleuven.be (V.V.A.); bart.vanrumste@kuleuven.be (B.V.); 2 KU Leuven, Stadius, Department of Electrical Engineering, 3001 Leuven, Belgium; 3 KU Leuven, HCI, Department of Computer Science, 3001 Leuven, Belgium; 4 LaSIGE, Departamento de Informática, Faculdade de Ciências, Universidade de Lisboa, Campo Grande, 1749-016 Lisboa, Portugal; jrocoelho@fc.ul.pt

**Keywords:** older adults, activity recognition, bathroom activities, wearable sensors, machine learning techniques

## Abstract

This article provides a systematic review of studies on recognising bathroom activities in older adults using wearable sensors. Bathroom activities are an important part of Activities of Daily Living (ADL). The performance on ADL activities is used to predict the ability of older adults to live independently. This paper aims to provide an overview of the studied bathroom activities, the wearable sensors used, different applied methodologies and the tested activity recognition techniques. Six databases were screened up to March 2020, based on four categories of keywords: older adults, activity recognition, bathroom activities and wearable sensors. In total, 4262 unique papers were found, of which only seven met the inclusion criteria. This small number shows that few studies have been conducted in this field. Therefore, in addition, this critical review resulted in several recommendations for future studies. In particular, we recommend to (1) study complex bathroom activities, including multiple movements; (2) recruit participants, especially the target population; (3) conduct both lab and real-life experiments; (4) investigate the optimal number and positions of wearable sensors; (5) choose a suitable annotation method; (6) investigate deep learning models; (7) evaluate the generality of classifiers; and (8) investigate both detection and quality performance of an activity.

## 1. Introduction

Globally, the population is ageing rapidly. By 2050, the number of people aged 60 years and older is expected to double to 2.1 billion [1]. This ageing process introduces physical and social challenges for older adults [2,3,4,5]. One prominent element is the decreased ability of performing Activities of Daily Living (ADL), which affects older adults’ ability to live independently at home. In the past years, a variety of devices have been used and researched to overcome limitations related to ageing and to help older adults stay independently at home, including wearable sensors, cameras, audio recorders and Internet of Things devices [6,7,8,9,10]. These devices can be used for recognising specific situations and activities in the daily life of older adults. Additionally, such devices can also be extended with functionalities that alert clinical staff, caregivers or trusted family members when a suspicious situation occurs [11,12,13]. Wearable sensors in particular, mainly accelerometers, have already been widely applied for the monitoring daily activities [12,14,15]. Compared to cameras and audio recorders, wearable sensors are superior in the number of recognised activities [10] and the protection of privacy and security of users. Compared to environmental sensors (presence, pressure, temperature etc.), wearable sensors can detect more complex activities rather than only one action. For example, the water flow detection sensor attached to the sink can detect the flow of water. However, it cannot detect whether the person is washing their hands or washing their face. To detect an activity, it should be combined with other sensors, which increases the complexity of device set-up. Compared to radar sensors, wearable sensors are not limited by the customised hardware set up and environmental interference [16].

The performance on ADL is usually assessed to predict the ability of an older adult to live independently [17,18] and to indicate functional frailty. For example, the Katz Index of Independence in Activities of Daily Living, is a scale to measure six ADLs (bathing, dressing, toileting, transferring, feeding and continence). The performance score is evaluated based on whether the participant can perform activities by themselves or not [19]. The final scores are then used by care providers to estimate care needs and by care planners for the distribution of health care resources [20]. Additionally, the performance quality on ADL can reveal the functional status of older adults, which can form the basis for therapeutic decisions, particularly when applied to certain diseases that are associated with old age, e.g., chronic obstructive pulmonary disease [21] and stroke [22].

ADLs can be categorised into different ways, but the most researched and monitored group is composed of the basic ADL (BADL). These BADLs involve bathing, grooming, maintaining dental hygiene, nail and hair care, dressing, toileting, continence, walking and feeding [17]. Among these BADLs, bathroom activities take up a large ratio. Bathroom activities are prominent in taking care of personal hygiene and preventing the spread of communicable disease [23].

However, to the best of the authors’ knowledge, few studies have been conducted on bathroom activity monitoring for older adults, especially using wearable sensors [6,7,9,24,25,26,27]. Therefore, the objective of this study is to review such studies and examine the actual studied bathroom activities, used wearable sensors, different applied methodologies and the activity recognition techniques. From this review, recommendations are generated for future studies.

This article presents a systematic review study of related work surrounding the field of bathroom activity recognition in older adults using wearable sensors. Literature encompassing conferences and journal papers were investigated to not only build the state-of-the-art but also lay the ground for recommendations as well.

This systematic review focuses particularly on the following research questions (RQ):

RQ1. Which **bathroom activities** are explored in current studies?
RQ2. Which **wearable sensors** are used?
RQ3. Which **methodology**, regarding the setup of the experiment, the number and type of participants, and the method of annotating the activities, is used in the current experiments?
RQ4. Which **activity recognition techniques** are investigated for bathroom activities?

To this end, Section 2 presents the methodology for selecting the search query and therefore including all relevant papers. Next, the retained studies are presented individually in Section 3, followed by a discussion in Section 4 where all research questions are answered. Additionally, at the end of each research question, recommendations are proposed to researchers who want to perform activity recognition in bathroom activities for older adults. A summary on the conducted systematic review is given in Section 5.

## 2. Methods

**Figure 1:**
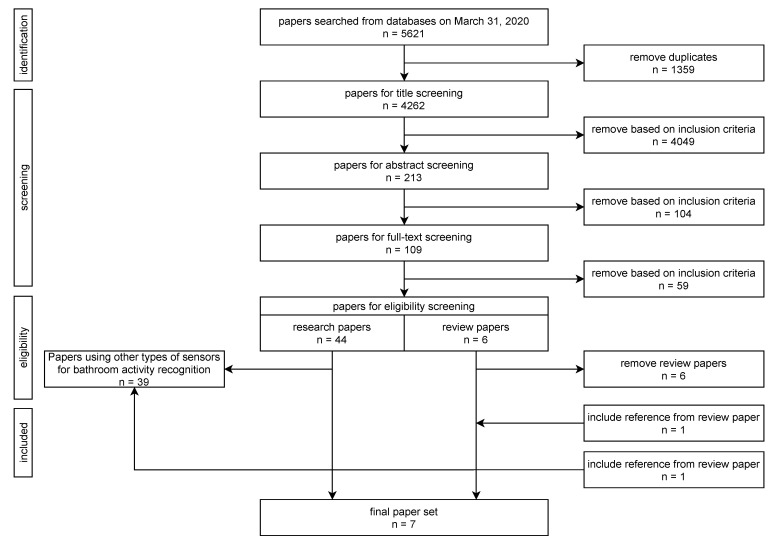
Flowchart of the paper screening procedure.

The focus of this review is on bathroom activities, such as bathing, showering, (un-)dressing, grooming, toileting and brushing teeth, recognised via wearable sensors. The protocol for searching and selecting papers was according to the recommended PRISMA guidelines on preferred reporting items for systematic reviews and meta-analyses [28], schematically represented in Figure 1.

### 2.1. Search Query

In order to find all relevant studies, keywords were organised into four categories, classified by concept; i.e., older adults as target population, activity recognition as outcome, bathroom activities as specification, and wearable sensors as materials. In two iterations, the researchers Y.Z, I.D, and J.C (also co-authors of the paper) identified and discussed relevant synonyms for these keywords. As only few papers were found in a pilot test, search criteria were expanded in order to not miss any relevant studies. The final set can be found in Table 1. By combining these concepts with the logical AND operator, the final query was formed.

Papers were searched in six libraries, both technological and general: IEEE Xplore, Science Direct, PubMed, Scopus, Web of Science and ACM. The keywords searching fields covered title, keyword and abstract. This search included all relevant papers, which were published in English until March 2020. Some libraries were limited in their advanced search feature, e.g., max. 15 search terms (IEEE), max. 8 logical operators (Science Direct, ACM) or only 2000 results to download (Scopus). Therefore, depending on the limitations of the library, smaller subcategories were generated to create and retrieve all possible combinations. Note that these combinations for all search terms were generated via a coded computer program to avoid mistakes and forgotten combinations. The subcategories of these keywords can be found in Appendix A.

### 2.2. Inclusion and Exclusion Criteria

To be included in the study, the paper should focus on older adults as the target population, but the age of participants was not limited. Papers should focus on at least one bathroom activity, i.e., bathing, showering, (un-)dressing, grooming, toileting or brushing teeth. In addition, at least one wearable sensor, either worn on the body or in pockets, should be used to recognise activities. Studies that included other activities or additionally implemented different sensors were retained at first. Afterwards, a distinction was made between wearable sensors and other sensors, in order to gain insights into these different approaches. However, the final analysis was limited to papers using wearable sensors. In addition, all papers needed to be published in English and should be of sufficient length to warrant a scientific contribution, e.g., at least 1500 words. Notes or one-page papers were excluded.

### 2.3. Search Procedure

As a result of querying in the six different libraries, 5621 papers were found. Zotero [29] was used as the management tool. An overview of the screening procedure is shown in Figure 1. Due to the aforementioned need of performing different searches and combinations, many duplicates were found, with a total of 1359 duplicated papers being excluded. The remaining 4262 papers were divided over three researchers to be further investigated. Papers were screened at first on titles and 4049 papers were excluded. This large number is due to the fact that Web of Science also includes “Keywords Plus^®^” [30], i.e., extra keywords based on references used in the paper, and also lemmatisation, i.e., including related words, often used in libraries. By these broader search terms, we wanted to guarantee that all relevant papers were included. However, this also implied finding a big number of papers to exclude based on a title screening. For example, *aged* or *old* are often used to describe participants’ age in general, and also *hygiene* was often found related to “oral hygiene”, e.g., “Caries Increment and Oral *Hygiene* Changes in 6- and 12-Year-*Old* Children”.

Next, papers were screened on the abstract (n = 213) and, afterwards, the entire paper was read (n = 109). When in doubt, the papers were discussed with all three researchers before including or excluding them, resulting in 50 papers. Review papers were initially retained and screened [31,32,33], making sure that all relevant papers mentioned by these studies were included as well. Based on this screening, two papers [9,34] on activity recognition of older adults were added. These two papers were not included in our initial findings as they did not cover all the keywords from the four concepts in the searching filed. The paper in [9] did not include the keywords of “older adults” and the paper in [34] did not include the keywords of “bathroom activity”.

**Figure 2:**
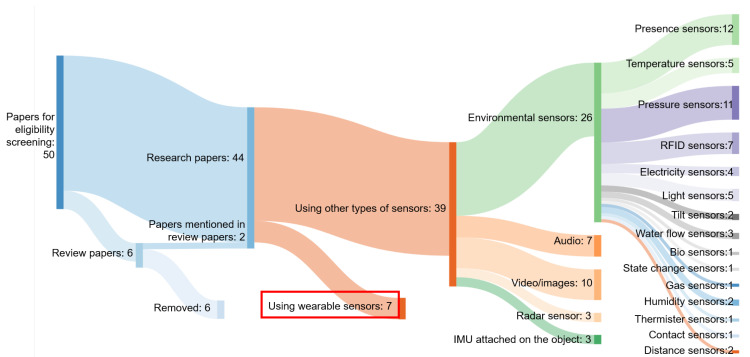
Sankey Diagram of the overview of the papers for eligibility screening.

Finally, papers were excluded based on eligibility, removing an additional 39 papers. The eligibility screening process is illustrated in Figure 2. Among the 50 papers, six papers were literature review papers. The other 44 papers were research papers, including bathroom activities recognition for older adults, using wearable sensors or other types of the sensors. Two extra research papers were included which were mentioned from the review papers. Thus, in total 46 research papers were screened for eligibility.

Among these 46 papers, studies in seven papers used wearable sensors. The other 39 papers used other types of sensors, among which audio recorders, video/image recorders, radar sensors, inertial measurement unit (IMU) sensors (accelerometer and/or gyroscope) attached to the object instead of the human body and environmental sensors. Audio recorders were used for detecting activities related to water flowing, e.g., washing hands [35,36,37,38,39], washing face [36], toileting [38,40,41] or bathing [40]. Video/image recorders were usually used for detecting washing hands [35,39,42,43,44], washing face [36] and dressing [45,46,47,48]. Radar sensors were used for detecting standing/sitting while showering [49], entering the bathtub [49,50] and sitting on/leaving toilet [51]. IMU sensors were attached to the water pipe for detecting washing and bathing [52], on the robot for detecting dressing [46], and in the soap bar for hand washing [37]. Multiple environmental sensors were combined for detecting bathing/showering [34,36,40,53,54,55,56,57,58,59], toileting [34,36,38,40,41,55,56,57,58,60,61,62,63,64,65,66,67,68,69,70], dressing [45,58,71,72], general personal hygiene activity [54,61], washing hands [36,37,38], washing face [36] and grooming [58,70].

Compared with wearable sensors, other types of sensors have limitations for detecting bathroom activities. First, the bathroom setting is a private environment, especially for activities like bathing, dressing and toileting. Thus, videos or images should be handled with the utmost care to protect the privacy of the users [34,36]. Second, using only an environmental sensor or audio recordings, the number of detectable activities are limited. As afore-described, environmental sensors usually only detect one action, which is not useful for detecting complex activities of daily living in the bathroom, which include a sequence of multiple actions [36,39,53]. Audio sensors can detect activities that create typical sounds, like washing hands [36,39]. However, it is limited in detecting dressing and combing hair, and it is noise-sensitive [52,56]. Therefore, with an increased number of activities that need to be detected, the number of sensors also increases. These reasons motivate the choice to focus on wearable devices for bathroom activity monitoring.

In conclusion, a final set of seven papers were studied in depth. All three authors analysed the selected studies and extracted data regarding target population, activities, sensor use, participants, setup of the experiment, feature extraction and recognition techniques. An overview of this information is shown in Table 2 and Table 3.

## 3. Results

Results are presented in Table 2 and Table 3. Below, the studies of seven papers will be discussed individually, addressing the aforementioned research questions.

Chan et al. [6] focused on two bathroom activities: washing hands and toileting (urination and defecation). Washing hands was included in this study as it was part of the toileting event. To collect movement signals, two accelerometers were used: one attached to the right wrist and the other one attached to the waist of the participant. Detailed information about the sensors was not mentioned, except its sampling rate at 20 Hz. In addition, little information was given about the experimental setup, neither the number of participants nor their age. They only reported that the toileting activity was performed 50 times. The collected signals were directly transmitted to the smartphone via Bluetooth. The signals were segmented by a sliding window at the length of 2 s, with 97.5% overlap. A two-layer hierarchical model was trained for recognising the activities, with 10-fold cross-validation. The first layer was used to classify segments, with six handcrafted features as input, all listed in Table 4. Four classifiers were tested: reduced error pruning decision tree (REP-tree), sequential minimal optimisation (SMO), random forest (RF) and Naive Bayes forest. Regarding the computation cost and accuracy result, the REP-tree scored best, achieving an overall accuracy of 76%. The classification result of the first layer was later used as the input of the second layer to predict the activity sequence. In the second layer, researchers developed a variable ordered hidden Markov model (VOHMM). Authors verified that compared to the traditional HMM model, this model is effective when an unreasonable class of segment is predicted [6]. For instance, in a sequence of segments for defecation, the first segment was predicted as the state of defecation but the last one was predicted incorrectly. To solve this problem, VOHMM defines a fixed order with excluding the possibility of transition to the unreasonable states. In other words, the current state determines which were the next possible states. As a result, VOHMM obtained the overall accuracy of 73%, around 43% higher than traditional HMM. In a future study, Chan et al. [6] proposed to study an adaptive model which can simplify the training process.

The study by Cherian et al. [7] did not only focus on the older adults, but also people with dementia. However, it is unclear which groups were recruited in this study. The studied ADLs included brushing teeth, drinking, combing hair, washing hands, scratching the chin and taking medicine. To collect the movement signal, a Pebble smartwatch was used, which was integrated with an accelerometer. Although the sensor position was not mentioned specifically, given that the study focused on detecting brushing teeth, it can be derived that the sensor was worn on the dominant wrist. The sampling rate was 25 Hz and the data were transmitted via Bluetooth to a smartphone. This study was conducted in three phases: recognising fixed ADLs, recognising brushing teeth within ADLs in a lab environment, and real-life environment. Respectively, 20, 6 and 12 unique participants (age unknown) were recruited in the three phases. During the first two phases, the activities were annotated by researchers and, in the third phase, it was done by the participants. The obtained signal was segmented by a 4-second sliding window, with 50% overlap and then processed to extract features. From each axis of the acceleration, 17 types of features were extracted, including seven types that were exclusively related to brushing teeth movements, as shown in italic in Table 4. A Correlation-based Feature Selection (Cfs) Subset Evaluator in WEKA [73] was used to search the optimal feature subset where each feature was highly correlated with the class and not correlated with each other. Five traditional machine learning techniques were investigated: C4.5 decision tree, k-nearest neighbors (KNN), multilayer perception, random tree (RT) and RF. In phase 1, the classifiers were trained in 10-fold cross-validation. RF reached the highest result, with an accuracy of 93.5% for brushing teeth, 64.0% for combing hair and 75.1% for washing hands. In phase 2, the model was trained for binary classification with brushing teeth versus the other five activities. To solve the imbalance problem, data collected in phase 1 and 2 were mixed together. Finally, C4.5 obtained the best result, with the averaged accuracy of 96.1% and F1-score of 0.620. Phase 3 also used binary classification, with the model trained using the data of phase 1 and 2 and tested on the data of phase 3. In this phase, C4.5 still obtained the best result, with an accuracy of 93.6% and F1-score of 0.824. Similar to the study by Chan et al. [6], the segment prediction result was used for predicting the sequence of brushing teeth. Three threshold values were set up: minimum brushing teeth duration (60 s), maximum pausing duration while brushing teeth (15 s) and ratio of brushing teeth duration taking up the total sequence duration (75%). As a result, 94% of brushing teeth sequences were detected. Concerning future studies, Cherian et al. [7] suggested to test the model in a real-life environment and generalise the model to predict other activities, like washing hands and taking medicine, as well. The final aim in the long run would be a personalised intervention system.

Noury et al. [24] focused on eight representative ADLs, including toileting and dressing. Besides a lab-developed actimometer (integrated with three accelerometers), a PIR detector and one central clock were also used. The actimometer was attached to the chest, with an unknown sampling rate. The experiment was conducted in a real-life setting of a flat. Seven younger participants (mean age: 27 years old) and four older participants (mean age: 80.5 years old) were recruited. Participants were asked to annotate the activities by themselves. After being segmented with 15-second windows, collected signals were processed to generalise six features: posture (lying, standing or sitting), transfers (sitting down, standing up or none), static/dynamic activity, walking or not, location (room) and time (hour). The features were fused together to calculate the Bayesian posterior probability. Final results of different populations, younger and older participants, were calculated separately. Compared to younger participants, the overall classification results of older participants were higher, with a recall (ratio of positive samples correctly predicted) of 86.9% versus 67.0%, and the specificity (ratio of negative samples correctly predicted) of 59.3% versus 52.6%. Considering toileting and dressing, the group of older participants also achieved better classification results. For toileting, the recall was 100% for older participants, compared to 77.7% for younger participants. The specificity was 92.8% and 85.0% for older and younger participants, respectively. The results for dressing, however, were much lower than those for toileting. The recall of dressing was 33.3% for older participants and 23.0% for younger participant. The specificity was 18.9% for older participants, which was 1% higher than that for younger participants. To improve the performance of the system in future studies, researchers proposed to recruit more participants, especially older adults. In addition, performing a longer experiment and using Beacon trademarks to personalise the location and time information while detecting multiple people simultaneously were also proposed to improve the results.

Different from the aforementioned studies, instead of detecting multiple activities, Kim et al. [25] aimed to monitor brushing teeth only and develop a training system. To accomplish this, three stroke patterns of brushing teeth movements were predicted: vertical, left-right and up-down. Each stroke pattern was further divided into three semi-patterns based on the teeth position: the front, left molar and right molar. One accelerometer and one magnetometer were integrated in the tooth brush for collecting signals. Data were sampled at a rate of 50 Hz and transmitted via radio frequency wireless communication. Four participants (mean age: 25 years old) were recruited. During the experiment, an application was used to show participants a 3D render of their tooth brushing movements. Detailed information about the experiment’s duration was not reported. The patterns were estimated using the processed data of the accelerometer and the magnetometer, together with predefined threshold values. The predicted patterns of each participant were eventually compared with the 3D render patterns to calculate the accuracy (coincidence rate). Among the participants, the highest accuracy reached to around 83% and the lowest one reached to around 72%. In the future, researchers would like to observe for more brushing teeth patterns and explore which patterns are most suitable for correctly, in terms of quality, brushing your teeth.

Garlant et al. [26] performed both a qualitative and quantitative analysis to determine the similarity of the movement signals of activities, inter- and intra-subject. This contrasts with the other studies that mainly focused on classifying activities. In this study, four participants, aged 22–24 years old, were recruited in the experiment. They were asked to perform activities by following the instructions of researchers. Four ADLs were investigated, including one bathroom activity: brushing teeth. During the experiment, participants were asked to take the toothbrush using their dominant hands, pretend placing the toothpaste using the other hands and brush teeth for 30 s. The activities were annotated by the researchers. One BiostamRC${}^{TM}$ [74] sensor was attached to the dominant hand, with the sampling rate at 100 Hz. This sensor consisted of one accelerometer and one gyroscope. The collected signals were transmitted via Bluetooth to a tablet. The raw signals of both the accelerometer and gyroscope were used for analysis without processing. In the qualitative analysis, ten volunteers were asked to match the unlabelled signals with the labelled signals by observation. The similarity analysis of the same activity among participants were conducted in two ways: (1) *semi-quantitative*: evaluating the similarity degree between participants via calculating the overall congruence of signal envelope. The similarity was graded from 1–5, with 5 presenting for total overlap; (2) *quantitative*: calculating the Pearson correlation between participants. The result showed that brushing teeth patterns were subjectively different, with an overlapping score of 2 and low correlation values ranging from r=0.62 to r=0.68. In future studies, Garlant et al. [26] aspired to focus on automatically recognising activities; next to manual and statistical methods, machine learning techniques should be explored. Moreover, they argued that other physiological sensors, like ECG, should also be used.

Masum et al. [27] mainly focused on basic physical activities, including toileting. A Xiaomi Redmi 4A smartphone was attached to the hand, with data collected at a rate of 1 Hz. In total, 20,000 activities—walking (upstairs/downstairs), sitting, standing, lying, toileting, writing and typing—were recorded in both male and female participants (ages and numbers are unknown). For training the model, the raw acceleration and gyroscope signals were directly used as features. Principal Component Analysis (PCA) was used to analyse the features, and the result implied that, compared to only using the signal of one sensor, the combination of these two sensors is better in classifying activities. Support vector machine (SVM), decision tree, KNN, RF, Naive Bayes (NB) and logistic regression (LR) were used as classifiers. In addition, a Dense Neural Network (DNN), with six hidden layers, was also investigated. The number of neurons of each hidden layer used were respectively 500, 500, 500, 300, 200 and 100. The classifier was trained at the learning rate of 0.0001, with the maximum iterations of 2000. This study was also aimed to investigate the classification difference between genders. Thus, 80% of the data of each gender were taken out and mixed together for training the data. The classifiers were tested on the remaining 20% of the data, for each gender group separately. The authors found the DNN model outperformed other algorithms, with the highest accuracy both in female (94.38%) and male (93.35%) data set. The accuracy results of RF were about 1% lower than the DNN model. This could be because that the nested hierarchical architecture of the DNN model can extract more complex features by nonlinear conversion of the input data. For toileting, the data set of male participants obtained an accuracy of 99.7%, and the data set of female participants obtained 100%, which implied that there was no significant difference between genders. Considering the performance of DNN, researchers expressed they would like to investigate more deep learning models in the future.

De et al. [9] aimed to use activity recognition to track the progression of certain diseases, e.g., Alzheimer. Among the studied activities, two bathroom activities were included: toileting and using a bathroom sink. The researchers did not specify the activity related to using a bathroom sink, thus it could equally be washing hands, washing the face, etc. In this study, four Galaxy Sr smartphones were used simultaneously, placed on the waist, lower back, thigh and the wrist separately. One software application was developed for supervisors to annotate the activity. Only one person participated in the experiment, demographics unknown. The participant performed activities in his/her preferred order for two iterations, each iterations for 45 min. The data collected from one iteration were used as training data set, and the other iteration’s as data set. Signals were collected by an accelerometer, a gyroscope, thermometer, humidity sensor and atmospheric pressure sensor, which were available in the smartphones. Note that features from acceleration or gyroscope were not extracted from each axis, but their magnitude ($Mag=\sqrt{Ax^{2}+Ay^{2}+Az^{2}}$). A Conditional Random Field (CRF) model was applied as the classifier. The signals of the four smartphones were fused at the decision level. The final recognition result was determined by the results of the sensor of which the attached body position was most relevant to the performed activity. Finally, using a bathroom sink and toileting achieved an accuracy of 75% and 95%, respectively.

## 4. Discussion

This section revisits the four research questions, as articulated at the end of Section 1. Considering that only seven studies were found, despite the widely cast net in querying the libraries, future studies on bathroom activity recognition in older adults by using wearable sensors is needed. Therefore, for each research question, recommendations are formulated, based on the lessons learned from studies in this systematic review.

### 4.1. RQ1. Which **Bathroom Activities** Have Already Been Explored in Current Studies?

In total, seven distinct bathroom activities were investigated, which can be listed according to the number of times they were included: toileting [6,9,24,27], brushing teeth [7,25,26], washing hands [6,7], combing hair [7], dressing [24] and using a bathroom sink [9]. Therefore, this review study shows that most frequently studied bathroom activities are those characterised by simple movements, like toileting, or those characterised by periodic movements, like brushing teeth. Such activities achieved relatively high classification performance as well. Bathroom activities like bathing or showering were not found to be studied using wearable sensors, and other bathroom activities like washing hands or dressing still obtained a low classification result. Currently most studies focused on classifying certain activities, whereas, in a real-life application, it is recommended to detect these activities among other ADLs as well. Besides detecting activities, especially as a prediction the live independently ability of older adults, the quality of performing these activities should be taken into account as well.

Therefore, based on the activities that are studied in the current state of the art, researchers who aim to extend the state-of-the-art should:

- **Investigate more types of bathroom activities.** Dressing, showering, and bathing should be given priority in the future study.
- **Not only classify, but also detect activities.** Perform real-life experiments where activities can be detected among all other daily actions.
- **Evaluate the activity performance quality.** Besides classifying or detecting an activity, one should also investigate whether this activity is performed as desired.

### 4.2. RQ2. Which **Wearable Sensors** Were Applied?

In this systematic review, the focus was on wearable sensors, attached to different body positions. Sensors were attached to the participant’s hands [25,26], to the wrist [6,9], to the waist [6,9], to the chest [7], to the lower back [9] and to the thigh [9]. One study integrated the sensors in a toothbrush, as it was only focused on different types of brushing teeth patterns [27].

For washing hands, brushing teeth and combing hair, wearable sensors were attached to the wrist [6,75], to the hand [26] or integrated in the toothbrush [25]. For toileting, the attached body parts varied from the wrist [6,9], to the waist [6,9], the chest [15], the hand [27], the lower back [9] and the thigh [2]. Note that for every activity measured, the performance of the machine learning algorithm is highly dependent on the position of the sensor on the body.

Combining multiple sensors can improve the recognition accuracy of activities [76]. In De et al. [9], the classification results of the fusion of four smartphones were better than only using one smartphone. However, more sensors increases the complexity of the system. As a result, installation and maintenance workload will increase. The user acceptance rate is also influenced by the type and number of sensors [8]. Park et al. [8] state that especially for environmental sensors, compatibility with the traditional devices, connectedness and cost are prominent factors for user acceptance.

Compared to other wearable sensors, the use of a smartphone proved to be more likely to support tracking activities for a longer time-span [77]. This could be due to the fact that participants often already have a smartphone, and therefore do not need to get familiar with using the sensors. Among the seven studies, two studies [9,27] made use of the smartphone and verified its feasibility. However, current studies did not mention the orientations (e.g., landscape or portrait) of the sensors, which could impact the algorithms.

Regarding devices, many different wearable sensors were used. Below, two recommendations for choosing a device in future studies are presented.

- **Combine accelerometer with other types of sensors.** When activities have similar movements like washing hands, or washing food, environmental or physiological sensors can be used for detecting (previously conducted) related activities.
- **Investigate the optimal attachment position of the wearable sensor.** As aforementioned, the position of the wearable sensors on the human body is tightly related to the studied activity. Future studies should further investigate the influence of different positions and orientations of the devices on activity classification.

### 4.3. RQ3. Which **Methodology**, Regarding the Setup of the Experiment and Annotating the Activities, Was Used in the Current Experiments?

In five of the seven included studies, the experimental procedures were not described in detail [6,9,25,26,27]. Therefore, it is hard to evaluate their experimental protocol and impossible for others to repeat their experiments, making it hard to generalise results.

Three studies (Kim et al. [25], Garlant et al. [26], Masum et al. [27]) conducted lab experiments, and two studies (De et al. [9], Noury et al. [24]) performed real-life experiments in a living lab. Although a living lab may increase ecological validity, we found natural influences to be still limited. For example, there were no disturbances, such as a phone call, to interrupt and add noise. Therefore, an extra study in a real-life environment would be necessary to also investigate real-world, noisy signals. The abilities of classifiers in real daily living situations remain unknown.

In addition, the experiment period was short and included few participants, often less than ten participants. Thus, the classification model training was impacted, like the overfitting problem faced in the study by Chan et al. [6]. From the perspective of recruited participants, even though all the studies included older adults as their target population, only Noury et al. [24] reported to include older adults as participants. Considering the performance difference between older and younger adults in the movement speed and amplitude, the results of the other six studies are difficult to be generalised to the older adults. Below, four recommendations are provided for performing experiment.

- **Conduct experiments with participants in three phases.** First, a controlled lab experiment is necessary to test the protocol and the sensor setup. Afterwards, an uncontrolled lab environment, or living lab, could help to pilot the study. However, finally, a study in the participants’ own living environment is necessary to generalise results. Participants will probably act different without any supervision, which are important actions to validate algorithms.
- **Increase the diversity of participants and ensure participants are representative for the target population.** It is strongly recommended to include older adults as participants. In addition, as the frailty status will impact the activity performance, not only healthy older adults, but also participants at different frailty levels need to be studied.
- **Conduct a longer period experiment.** By conducting experiments with participants during several weeks or months, the performing patterns of activities, like frequency, duration and performing time, can be monitored. Moreover, the change of the patterns can be used to predict the decrease/increase of the living independently ability of older adults.
- **Select an applicable annotation method.** This is important in order to acquire high quality data from the experiment, to be used as a ground-truth. Based on the type of experiment (controlled, uncontrolled or real-life), a researcher could use a protocol to annotate for the participants, let participants annotate by themselves, or consider ways to automate annotation through a third system. Depending on the activity, privacy may be an issue, e.g., installing a camera in the bathroom. When participants need to annotate by themselves, this method should be made as easy as possible. Forms of annotations include a diary, checklist, software application or automatic annotations by PIR sensors. Note that software applications are often preferred by researchers, but difficult for older adults to handle, as they need to get familiar with it. In addition, be careful with participant-made annotations that function as ground truth. These may contain errors too.

### 4.4. RQ4. Which **Activity Recognition Techniques** Were Investigated for Bathroom Activities?

In total, three different types of models were applied: statistical models, machine learning models and deep learning models. However, it was hard to compare the performance between the different studies, as they were tested on different data sets. For the method of model training, only one study discussed the generalisation ability of the classifier for new participants [7]. Others first mixed the data from different participants together, then took out part of the mixed data set for training. While training the model, imbalanced data could affect the evaluation of the performance of the model, as the model will be trained to reach a higher performance for the classes with more data points. From the seven papers, only Cherian et al. [7] tried to solve the imbalanced problem by combining the data of two phases together to train the model. The most applied evaluation metrics were accuracy [6,7,25,27], precision [27], recall [24], specificity [24], and F1-score [7]. In an unbalanced data set, it is common to use an F1-score instead of other metrics, as it combines the result of precision and recall and does not rely on true negatives [78].

Usually, regarding the signal preprocessing approach, filtering techniques with band- or low-pass filters are used to remove noise and extract movement components of the acceleration signals [79,80,81]. Curiously, none of the included studies applied these techniques. This may suggest that such filtering techniques are not needed for bathroom activity recognition. In the studies which used machine learning techniques [31], the features were extracted from the time domain and/or frequency domain, or directly the raw signals were used as features. In addition, in [7], new handcrafted features were extracted for detecting brushing teeth specifically.

As many different techniques can be used to detect activities, based on the results, the following recommendations are proposed:

- **Balance data set.** An imbalanced data set can affect the classification performance of the class taking up the smaller ratio. To solve this problem, multiple resampling techniques can be applied: oversampling [82], undersampling [83,84] and hybrid method [85,86]. These are usually applied for handcrafted features. If raw signals are directly used as the input in deep learning models, the augmentation method can be further researched. Proposed methods are permutation, time-wrapping, scaling, jittering and cropping of the raw signals [75].
- **Generalise the classifiers.** To simulate real-life application, classifiers need to include new participants in the test data set. The method of leave-one-subject-out (LOSO) is suggested for training classifiers, with taking the data set of one subject out as the testing data set and the data set of the other subjects as training data set. In this situation, to overcome the impact of subjective difference, transfer learning can be applied, via taking part of the test subjects’ data set into the training data set.
- **Investigate deep learning classifiers.** Deep learning classifiers, like convolutional neural network (CNN), long short-term memory model (LSTM) and gated recurrent units (GRU), have already been widely applied in activity recognition using wearable sensors [13,87,88,89]. Compared to traditional machine learning algorithms, these classifiers can extract features directly from the raw signals without the need of determining and extracting handcrafted features. To understanding the meaning and the attribution of the extracted features, DeepExplain can be applied Ancona et al. [90]. As a large number of parameters need to be optimised in a deep learning classifier, massive data samples are needed. If there are limited samples, it is suggested to pre-train the classifier using a similar large data set, or augment the data set to increase the number sample.

### 4.5. Available Benchmark Data Sets

To the best of authors’ knowledge, the data sets of the seven papers have not been published. We searched for the relevant public data sets including bathroom activities with wearable sensors in five repositories, i.e., UCI [91], PhysioNet [92], Microsoft Research Open Data [93], Academic Torrents [94] and Figshare [95], and two search engines, i.e., Kaggle

(https://www.kaggle.com/datasets, accessed on 7 March 2021) and Google Dataset Search (https://datasetsearch.research.google.com/, accessed on 7 March 2021). As a result, four data sets [96,97,98,99] were found. The information about these four data sets is listed in Table 5. Papers related to these four data sets were not included in our review result, because they did not include keywords related to target population (older adults) and specification (bathroom activities) in the searching field. In the data set in [96,98], brushing teeth was included. The data was collected in the lab environment. In the data set in [97], grooming, dressing, toileting and bathing/showering were included. In the data set in [99], brushing teeth and showering were included. The data of these two data sets were collected in the real-life environment. However, there are still limitations of these four data sets, as they did not have older adults as their participants and they only included limited number of bathroom activities.

## 5. Conclusions

This study aimed to investigate the feasibility of recognising bathroom activities of older adults via wearable sensors. We surveyed current studies related to specific bathroom activities, wearable sensors, experimental setup and recognition techniques. While 50 papers were identified that focused on bathroom activities, only seven papers were found that uses wearable sensors, indicating that few studies have been conducted in this field. Moreover, from these seven papers, only one included the target population, older adults, as participants. Despite the limited studies found thus far, results suggest that it is possible to detect certain bathroom activities, such as brushing teeth, toileting and washing hands, using wearable sensors. Therefore, more studies need to be performed in the future, for which we generated recommendations, based on the review. We recommend that more complex bathroom activities can be investigated. Additionally, ideally a real-life experiment of a few weeks or months is conducted, including older adults as participants. Next, it is important to investigate the optimal combination of sensors and choose a suitable annotation method. Furthermore, we suggest to test more machine learning techniques, especially deep learning models, and also train the model using LOSO with transfer learning. Then, also the generality of the classifiers can be investigated. Finally, we recommend to investigate both detection and classification of activities, trying to account for a quality indicator of certain activities.

## Figures and Tables

**Table 1:** Selected keywords for search. An asterisk (*) was used as a wildcard to broaden the search for words starting or ending with a keyword.

Concept	Keywords
Target population: older adults	“older adult” OR “older person” OR “older people” OR elderly OR elder OR senior OR ageing OR aging OR aged OR retire* OR old OR gerontol* OR gerontechnology OR 60-plus
Outcome: activity recognition	monitor OR monitoring OR tele-monitoring OR user-activity OR recogni* OR detection OR detecting OR detect OR classif*
Specification: bathroom activities	“personal care” OR “wearing clothes” OR washing OR “brush* teeth” OR “teeth brush*” OR “brush* tooth” OR “tooth brush*” OR groom OR grooming OR “mouth care” OR bath OR bathing OR dress OR undress OR dressing OR undressing OR toilet OR OR toileting hygien* OR shower OR showering
Materials: wearable sensors	wearable OR sensor OR mobile* OR track* OR *worn OR portable OR monitor*

**Table 2:** Overview of extracted data from papers regarding target population, activities, sensor use, participants, setup of the experiment, feature extraction and recognition techniques.

		Target Population	Activities		Sensor Use					Participants	
Reference	Year		ADLs	Number of Bathroom ADLs	Wearable Sensors	Brands	Position	Frequency (Hz)	Additional Sensors	Mean Age (Years)	Number
Chan et al. [6]	2016	older participants, caregivers	**washing hands, toileting (urination, defecation)**	3	ACM	/	right wrist, waist	20	/	/	/
Cherian et al. [7]	2017	older adults, dementia patients	phase 1: drinking, **brushing teeth, combing hair, washing hands**, scratching the chin, taking medication phase 2,3: **brushing teeth**, others	phase 1: 3 phase 2,3: 1	ACM	Pebble	wrist	25	/	/	phase 1: 20 phase 2: 10 phase 3: 12
Noury et al. [24]	2012	older adults	walking, **dressing, toileting**, recreation, sleeping, breakfast, lunch, dinner	2	Actimometer (3 ACMs)	lab-developed	chest	/	/	27 (n = 7), 80.5 (n = 4)	11
Kim et al. [25]	2009	general (incl. older adults)	**brushing teeth**	1	ACM, MAG	ACM-MMA 7260, freescale, Tx; MAG-HMC 1055, Honeywell, MN	in toothbrush	50	/	25	4
Garlant et al. [26]	2018	general (incl. older adults)	walking, **brushing teeth**, drinking, from a mug, opening a pill bottle	1	ACM, GYR	BiostampRC	dominant hand	100	/	22–24	4
Masum et al. [27]	2019	general (incl. older adults)	walking upstairs, walking downstairs, walking, jogging, typing and writing, standing, sitting, lying, **toileting**	1	ACM, GYR	Xiaomi Redmi 4A smartphone	hand	data collection rate:1	/	/	/
De et al. [9]	2015	older adults, Alzheimer patients	using refrigerator, cleaning utensil, cooking, sitting and eating, **using bathroom sink**, moving from indoor to outdoor, moving from outdoor to indoor, walking upstairs, walking downstairs, standing, lying on the bed, sitting on the bed, lying on the floor, sitting on the floor, lying on the sofa, sitting on the sofa, and **toileting (sit on the toilet)**	2	ACM, GYR, temperature, humidity, atmospheric pressure	Galaxy S4 smartphone	waist, lower back, thigh, and wrist	ACM, GYR: 100, temperature, humidity: 1, atmospheric: 5	beacon trademarks	/	1

**Table 3:** Overview of extracted data from papers regarding target population, activities, sensor use, participants, setup of the experiment, feature extraction and recognition techniques (*continued)*.

	Setup of the Experiment			Feature Extraction			Recognition Techniques			
Reference	Test Period	Environment: Lab/Real-Life	Annotation Methods	Window Size	Number of Features	Domain	Machine Learning Algorithms	Other Algorithm	Evaluation Metrics	Model Training
Chan et al. [6]	50 times	/	/	40 data points, 95.5% overlap	6	time, frequency	REP-tree, SMO, RF, Naive Bayes forest, HMM, VOHMM	/	accuracy	10-fold cross validation
Cherian et al. [7]	phase 1: 79 min (total) phase 2: 1h per person phase 3: 4.9 h/6.25 days per person at least once per activity per day per person	phase1: lab phase2: real-life phase 3: real-life	phase1: supervisor phase2: participant phase3: participant,	4 s, 75% overlap	total: 51 after feature selection: phase 1: 24 phase 2,3: 13	time, frequency	C4.5, KNN, multilayer perception, RT, RF	/	accuracy, F1-score	phase1,2: 10-fold cross validation phase 3: LOPO
Noury et al. [24]	2 h in total	real-life	participants writing	15 s	/	/	/	self-developed method using probabilities	recall, specificity	/
Kim et al. [25]	/	lab	/	30 data points	/	/	/	threshold values	accuracy	/
Garlant et al. [26]	30 s for brushing teeth	lab	supervisor	/	/	/	/	statistical method	correlation, overlap rate	/
Masum et al. [27]	20,000 cases	lab	/	/	/	ACM, GYR	DNN, SVM, DT, KNN, RF, NB, LR	/	accuracy, precision	80% for training
De et al. [9]	90 min	real-life	supervisor (developed app)	2 s	ACM: 6 GYR: 6 temperature: 2 pressure: 2 humidity: 2 beacon trademarks: 1	time	/	multi-scale conditional random field	accuracy	50% for training

**Table 4:** Handcrafted features (time and frequency domain) for activity classification.

Reference	Signal	Features	
		Time Domain	Frequency Domain
Chan et al. [6]	acceleration	mean, standard deviation (std), correlation, signal magnitude area, tilt angle	spectral entropy
Cherian et al. [7]	acceleration	mean, std, mean jerk, mean distance between axes, correlation, number of peaks, number of valleys, root mean square, *std of valleys, mean of peaks, std of peaks, mean of peaks, mean of the side heights, std of the side heights, number of times axes crossed each other*	energy, entropy
Noury et al. [24]	acceleration, PIR signal, time	location, static/dynamic, walking or not, transfers, postures, time	/
Kim et al. [25]	acceleration, magnetometer	/	/
Garlant et al. [26]	acceleration, gyroscope	/	/
Masum et al. [27]	acceleration, gyroscope	raw signal	/
	magnitude of acceleration	mean, variance of the magnitude, mean, variance of the first derivative of the magnitude, mean, variance of the second derivative of the magnitude	/
De et al. [9]	magnitude of gyroscope	mean, variance of the magnitude, mean, variance of the first derivative of the magnitude, mean, variance of the second derivative of the magnitude	/
	temperature, humidity, atmospheric	subtle change	/
	beacon trademarks	location, presence	/

**Table 5:** Overview of the public data set regarding activities, sensor use, participants and setup of the experiment.

		Activities		Sensor Use			Participants		Setup of the Experiment		
Reference	Year	Bathroom ADLs	Number of Total ADLs	Wearable Sensors	Position	Frequency (Hz)	Age (Years)	Number	Average Test Period	Environment: Lab/Real-Life	Annotation Methods
Weiss et al. [96]	2019	brushing teeth	18	smartphone + smartwatch	smartphone-right pants pocket, watch-dominant hands	ACM, GYR: 20	18–25	51	3 min/ activity/ person	lab	/
Vaizman et al. [97]	2017	grooming, dressing, toileting, bathing/showering	51	smartphone + smartwatch	left wrist (none- dominant side for 93% of participants)	ACM, GYR, MAG (smartphone): 40, ACM (smartwatch): 25, audio: 22 k, location: recorded when the value changes, phone state: sampled once/activity example	18–42	60	7 days/ person	real-life	annotated by participants using software
Ruzzon et al. [98]	2020	Brushing teeth	nine	six IMUs (AcM, GYR)	left upper arm, left lower arm, right upper arm, fight lower arm, back, right thigh	33	22–28	10	16 min/person	lab	annotated by one researcher
Garcia-Ceja et al. [99]	2014	showering, brushing teeth	eight	smartwatch (ACM)	dominant wrist	20	/	2 (only 1 performed bathroom activities)	10.5 days/ person	real-life	annotated by participants by taking notes

## Data Availability

Not applicable.

## Appendix A. Keywords Subcategories of Libraries

### Appendix A.1. IEEE Explore

**Table A1:** Subcategories of Keywords applied in IEEE Explore. An asterisk (*) was used as a wildcard to broaden the search for words starting or ending with a keyword.

Concept	Keywords Subcategories
Target population:	P1: elderly OR elder OR senior OR ageing OR aging OR aged OR retire*
	P2: “older adult” OR “older person” OR “older people” OR old OR gerontol* OR gerontechnology OR 60-plus
Outcome:	O1 : monitor OR monitoring OR tele-monitoring OR user-activity OR recogni*
	O2: detection OR detecting OR detect OR classif*
Specification:	S1:personal care“ OR “wearing clothes” OR washing OR “brush* teeth”
	S2: “teeth brush*” OR groom OR grooming OR “mouth care”
	S3:“brush* tooth” OR bath OR bathing OR dress
	S4:undress OR dressing OR undressing OR “tooth brush*”
	S5: toilet OR toileting OR hygien*OR shower OR showering
Devices:	D1: wearable OR sensor OR mobile* OR track*
	D2: monitor* OR *worn OR portable

### Appendix A.2. Science Direct

**Table A2:** Subcategories of Keywords applied in Science Direct.

Concept	Keywords Subcategories
Target population:	P1: elderly OR elder
	P2: senior OR ageing
	P3: aged OR aging
	P4: old OR “older adults”
	P5: “older person” OR “older people”
	P6: 60-plus OR gerontechnology
	P7: retired OR retiree
	P8: gerontology
Outcome:	O1 : monitor OR monitoring OR tele-monitoring OR user-activity OR detection OR detecting
	O2: detect OR classifier OR classification OR recognition OR recognizing OR recognized
Specification:	S1: personal care“ OR “’wearing clothes” OR washing
	S2: groom OR grooming OR “mouth care”
	S3: bath OR bathing OR dress
	S4: undress OR dressing OR undressing
	S5: toilet OR toileting OR shower
	S6: showering OR brush OR brushing
	S7: teeth OR tooth OR hygiene
	S8: hygienic
Devices:	D1: wearable OR sensor OR portable OR mobile
	D2: tracking OR tracker OR monitor OR monitoring
	D3: monitorized OR monitored OR worn

### Appendix A.3. ACM

**Table A3:** Subcategories of Keywords applied in ACM. An asterisk (*) was used as a wildcard to broaden the search for words starting or ending with a keyword.

Concept	Keywords Subcategories
Target population:	P1: elderly OR elder OR senior OR ageing OR aging OR aged OR retire*
	P2: old OR “older adult” OR “older person” OR “older people” OR gerontol* OR 60-plus OR gerontechnology
Outcome:	O1: monitor OR monitoring OR tele-monitoring OR “user-activity” OR recogni*
	O2: detection OR detecting OR detect OR classif*
Specification:	S1: “personal care” OR “wearing clothes” OR washing OR “brush* teeth” OR “teeth brush*”
	S2: “brush* tooth” OR “tooth brush*” OR groom OR grooming OR “mouth care”
	S3: bath OR bathing OR dress OR undress OR dressing
	S4: undressing OR toilet OR hygien* OR shower OR showering
Devices:	D1: wearable OR sensor OR mobile* OR track*
	D2: *worn OR portable OR monitor*
